# Screening of a Specific Peptide Binding to VPAC1 Receptor from a Phage Display Peptide Library

**DOI:** 10.1371/journal.pone.0054264

**Published:** 2013-01-24

**Authors:** Bo Tang, Zhexu Li, Dingde Huang, Lei Zheng, Qianwei Li

**Affiliations:** Department of Nuclear Medicine, Southwest Hospital, Third Military Medical University, Chongqing, China; Univ of Bradford, United Kingdom

## Abstract

**Background/Purpose:**

The VPAC1 receptor, a member of the vasoactive intestinal peptide receptors (VIPRs), is overexpressed in the most frequently occurring malignant tumors and plays a major role in the progression and angiogenesis of a number of malignancies. Recently, phage display has become widely used for many applications, including ligand generation for targeted imaging, drug delivery and therapy. In this work, we developed a panning procedure using a phage display peptide library to select a peptide that specifically binds to the VPAC1 receptor to develop a novel targeted probe for molecular imaging and therapy.

**Methods:**

CHO-K1 cells stably expressing VPAC1 receptors (CHO-K1/VPAC1 cells) were used to select a VPAC1-binding peptide from a 12-mer phage peptide library. DNA sequencing and homologous analysis of the randomly selected phage clones were performed. A cellular ELISA was used to determine the most selectively binding peptide for further investigation. Binding specificity to the VPAC1 receptor was analyzed by competitive inhibition ELISA and flow cytometry. The binding ability of the selected peptide to CHO-K1/VPAC1 cells and colorectal cancer (CRC) cell lines was confirmed using fluorescence microscopy and flow cytometry.

**Results:**

A significant enrichment of phages that specifically bound to CHO-K1/VPAC1 cells was obtained after four rounds of panning. Of the selected phage clones, 16 out of 60 shared the same peptide sequence, GFRFGALHEYNS, which we termed the VP2 peptide. VP2 and vasoactive intestinal peptide (VIP) competitively bound to the VPAC1 receptor. More importantly, we confirmed that VP2 specifically bound to CHO-K1/VPAC1 cells and several CRC cell lines.

**Conclusion:**

Our results demonstrate that the VP2 peptide could specifically bind to VPAC1 receptor and several CRC cell lines. And VP2 peptide may be a potential candidate to be developed as a useful diagnostic molecular imaging probe for early detection of CRC.

## Introduction

Vasoactive intestinal peptide receptors (VIPRs), members of the G-protein-coupled receptor (GPCR) superfamily, are functional receptors for vasoactive intestinal peptide (VIP) and pituitary adenylate cyclase-activating polypeptide (PACAP). The VIPRs comprise three subtypes of receptors: VPAC1, VPAC2 and PAC1. VPAC1 and VPAC2 receptors share a common high affinity for VIP and PACAP, and PAC1 displays a high affinity for PACAP but a low affinity for VIP [1]–[2]. VIPR is characteristically activated via heterotrimeric G-proteins, resulting in AC activation, cAMP production, PKA (protein kinase A) pathway activation [1], [3], [4], and the stimulation of PKC (protein kinase C) [3], [5], PI3K (phosphatidylinositol 3-kinase [6], MAPKs (mitogen-activated protein kinases) [7]–[8] and NF-κB [9]. The effects of VIP and PACAP are mainly mediated through VPAC1 and VPAC2 receptors [1], [4], and they are involved in many physiological and pathophysiological processes, such as growth, cancer, immune responses, circadian rhythms, the control of neuronal and endocrine cells, and functions of the digestive, respiratory, reproductive and cardiovascular systems [10]. In normal human tissues, VPAC1 receptors are preferentially expressed in most epithelial tissues, while VPAC2 receptors are mainly expressed in smooth muscle tissue [11]. However, VIPRs are highly overexpressed in human tumors and their metastases. Similar to their expression pattern in normal tissues, VPAC1 receptors are overexpressed in frequently occurring malignant epithelial neoplasms, such as cancers of the colon, rectum, lung, breast, and prostate. In contrast to the ubiquitous expression of VPAC1 receptors in most human tumors, VPAC2 receptors predominate in a small subset of tumors, including leiomyomas and gastrointestinal stromal tumors [11], [12]. The difference in the cell surface profile between cancer cells and their normal counterparts can be utilized as a molecular signature for targeted imaging. Furthermore, the overexpressed VPAC1 receptors play a major role in the progression of a number of malignancies, including cancers of the lung, brain, gut, and prostate in addition to neuroblastomas [13], [14], and they mediate tumor angiogenesis through the transactivation of epidermal growth factor receptor (EGFR) and the expression of vascular endothelial growth factor (VEGF) [15], [16]. Thus, these data indicate that the VPAC1 receptor is a potential target for tumor diagnosis and therapy. The finding that most tumors predominantly express VPAC1 receptors at high levels has led to the development of in vivo imaging methods for the localization of certain types of tumors by targeting the VPAC1 receptor with radioactively labeled substances. Colorectal cancers (CRCs) are optimal tumors for targeting because of the relatively lower expression level of VPAC1 receptors in normal intestinal tract tissues compared with all other human tissues [11]. Thus, a higher tumor-to-background ratio can be obtained in CRC-targeted imaging and therapy. Therefore, the VPAC1 receptor is a potentially valuable target for the diagnosis and treatment of CRC, and the development of a specific molecular probe targeting the VPAC1 receptor would allow for early CRC detection and increased therapeutic efficacy.

Currently, the conventional noninvasive imaging diagnostic methods for the detection of new CRC lesions or changes in the size of a known lesion caused by cancer growth are computed tomography (CT) and magnetic resonance imaging (MRI) [17]. Even endoscopic techniques, which are the most sensitive conventional diagnostic methods, are limited in their sensitivity because the detection of CRC is limited to lesions the examiner can visualize [18]. Despite the widespread use of these conventional imaging modalities, their accuracy and sensitivity for the detection of CRC as well as recurrence and metastasis are relatively low. In view of this, the development of new methods that can sensitively detect CRC at earlier stages could have an important clinical impact. Fortunately, molecular imaging has provided a novel means of identifying and characterizing tumors and other lesions based on their protein expression pattern rather than their macroscopic morphology [19]. The molecular expression pattern of tumors such as CRC can be visualized with the help of tumor-specific molecular probes, such as peptides, antibodies and antibody fragments [20], [21]. Peptides appear to have advantages as detection probes for both imaging and targeting because of their smaller size, improved tissue penetration ability, shorter plasma half-life, and lower immunogenicity compared with antibodies [22], [23]. Therefore, the identification of novel peptides that bind to the VPAC1 receptor with high specificity and affinity is extremely important for the development of new peptide probes for the early detection and treatment of CRC.

Phage display is a molecular technology that allows the presentation of a large number of peptides or proteins on the surface of filamentous phage for various applications. The display of peptide libraries on the surface of bacteriophage permits the selection of peptides or proteins with high affinity and specificity for almost any target. Recently, the panning of phage display peptide libraries on intact cells in culture has proven successful for selecting peptides. All the peptide ligands tested showed high specificity and affinity for receptors both in vitro and in vivo; therefore, these peptide ligands may be useful for tumor-targeted diagnosis and treatment. [24]–[27].

In the present study, CHO-K1 cells stably expressing human VPAC1 receptors were used to screen a 12-mer phage display peptide library, and a novel peptide capable of specifically binding to the VPAC1 receptor was identified. Our results demonstrated that the peptide selected could compete with VIP for binding to the VPAC1 receptor and target colorectal cancer cell lines (HT29, SW480, and SW620). Thus, our findings suggest that the novel peptide identified in our experiments has great potential for use in the early diagnosis and treatment of CRC.

## Results

### Stable expression of the recombinant human VPAC1 receptor in CHO-K1 cells

**Figure 1:**
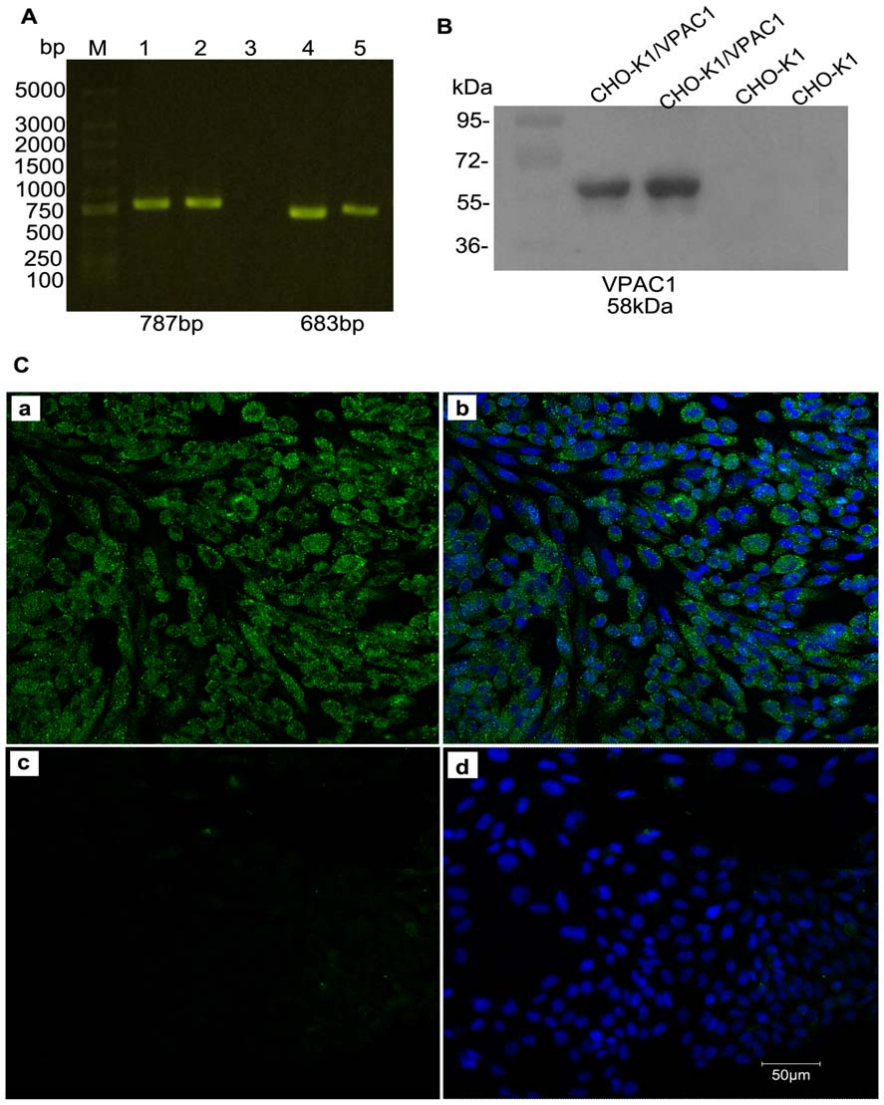
Stable expression of the recombinant human VPAC1 receptor in CHO-K1 cells. (A) Reverse transcription PCR of the VPAC1 gene expression. M: DNA marker DL 5000 bp, lane 1 and lane 2: VPAC1 gene expression in CHO-K1 cells transfected with pcDNA3.1(+)/VPAC1 plasmid, lane 3: VPAC1 gene expression in CHO-K1 cells, lane 4 and lane 5: GAPDH in CHO-K1 cells transfected and non-transfected with pcDNA3.1(+)/VPAC1 plasmid. (B) Western blot analysis of VPAC1 expression. Migration of molecular weight marker is indicated on the left of the blot. CHO-K1 cells transfected with pcDNA3.1(+)/VPAC1 plasmid yielded a single prominent band at approximately 58 kDa. CHO-K1 cells as a negative control. (C) Immumofluorescence analysis of VPAC1 expression. VPAC1 receptor was expressed on the cell membrane and accumulated in the cytoplasm of positive CHO-K1/VPAC1 cells (a), (b). CHO-K1 cells as the negative control (c), (d). (b), (d) represents the merged image.

We examined the expression of the VPAC1 receptor in transfected CHO-K1 cells using RT-PCR, immunofluorescence and western blot analysis. As shown in Figure 1, the VPAC1 gene could only be amplified from CHO-K1 cells transfected with the pcDNA3.1 (+)/VPAC1 plasmid (Figure 1A). Western blot analysis showed that the molecular weight of the expressed VPAC1 protein was approximately 58 kDa (Figure 1B), which was similar to that of full-length VPAC1. The immunofluorescence results indicated that the VPAC1 receptor was expressed on the cell membrane and accumulated in the cytoplasm. Additionally, we noted that VPAC1 was especially highly expressed in CHO-K1/VPAC1 cells compared with CHO-K1 cells (Figure 1C). Wild-type CHO-K1 cells were used as a negative control.

### Specific enrichment of CHO-K1/VPAC1 cell-bound phages

**Figure 2:**
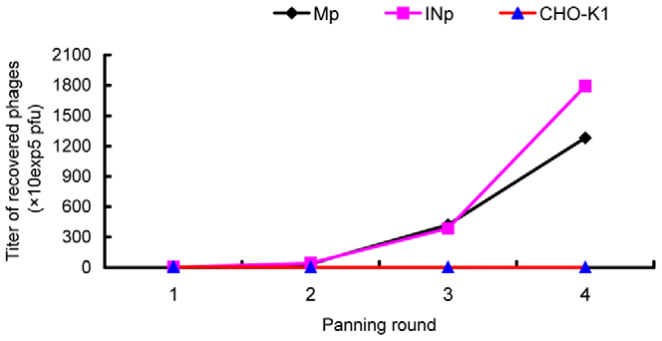
Specific enrichment of recovered phages. A specific enrichment of phages binding to CHO-K1/VPAC1 cells was seen after four rounds of panning. The titers of the recovered phages from each round were evaluated by the blue plaque-forming assay on LB/IPTG/X-gal plates. Here, Mp represents phages recovered from an acid elution fraction, INp represents phages recovered from a lysate fraction and CHO-K1 denotes phages recovered from CHO-K1 cells.

Phages that specifically bound to CHO-K1/VPAC1 cells were identified through four rounds of in vitro selection with CHO-K1/VPAC1 and CHO-K1 cells. In each round, the bound phages were rescued and amplified in ER2738 cells for the subsequent panning, whereas the unbound phages were removed by washing with TBST. After four rounds of panning, the titers of Mp and INp phages recovered from CHO-K1/VPAC1 cells were significantly increased by approximately 679.7 and 440.8 fold, respectively, compared with their titers in the first round. In contrast, the number of phages recovered from wild-type CHO-K1 cells remained at a low level and was even decreased after four rounds of panning (Figure 2). The output/input ratio of phages after each round of panning was used to determine the enrichment efficiency, which increased from 3.0×10^−6^ to 1.5×10^−3^ (Figure 2). These results indicated that phages that were capable of specifically binding to CHO-K1/VPAC1 cells were significantly enriched.

### DNA sequencing of the selected phage clones

After the fourth round of panning, 60 phage clones (20 each from Mp, Sp and INp) were randomly selected and sequenced, and the clones were designated Mp1–20, Sp21–40 and INp41–60. Three phage clones (Sp25, INp42 and INp55) lacked the exogenous sequence; however, the remaining clones were confirmed to be positive by DNA sequencing (Dataset S1). The deduced peptide sequences were analyzed and classified, and 18 different phage clones or peptide sequences were obtained. The peptide sequences of these 18 clones were designated VP1 to VP18, and VP2 appeared sixteen times (Table 1). Multiple sequence alignment analyses did not reveal strong homology among the different peptide sequences.

**Table 1:** Amino acid sequences of selected phage clones from 12-mer peptide library.

NO.	Phage clones	Peptide sequence	Frequency
VP1	Mp1-3/Mp7/Sp26/INp47-48/INp58	TVKYSTLVEWPY	8
VP2	Mp4/Mp6/Mp8-9/Mp13/Mp17/Mp19/Sp21/30/34/37/INp41/44/54/57/59	GFRFGALHEYNS	16
VP3	Mp5/Sp27	NSIALINDTHKR	2
VP4	Mp10/Mp20/Sp23	TWKFEPLGTFID	3
VP5	Mp11/Mp16/Sp24/Sp31/Sp40	DTFHSPLVALVS	5
VP6	Mp12/Mp14-15/Mp18/Sp32	YTSHFPLETWPQ	5
VP7	Sp22	ETVRQAEELFYV	1
VP8	Sp28/INp46/INp50/INp51/INp60	SFRFFPLDMWPH	5
VP9	Sp29	AYTTVPYMATLP	1
VP10	Sp33/INp45	GGSIAASELEYY	2
VP11	Sp35	HSTLKLGALTNY	1
VP12	Sp36	GSFHSPLLAYVS	1
VP13	Sp38	DTGHSPEPGKVP	1
VP14	Sp39	GTFHSPLLDHKS	1
VP15	INp43	TVTFAPLRMWHP	1
VP16	INp49/INp56	GWLRSPSLLFSN	2
VP17	INp52	TYTFRPLYEPPL	1
VP18	INp53	GYTFQPLNEWAI	1

After the fourth round of panning, 60 phage clones were randomly selected. The phage clones were sequenced and three phage clones (Sp25, INp42 and INp55) lacked the exogenous sequence. 18 different peptide sequences were obtained and designated VP1 to VP18. The frequency represents the number of the peptide sequence appeared in the whole selected phage clones. Here, Mp represents phages recovered from an acid elution fraction, Sp represents a specific elution by VIP, INp represents phages recovered from a lysate fraction.

### Confirmation of in vitro binding by cellular ELISA

**Figure 3:**
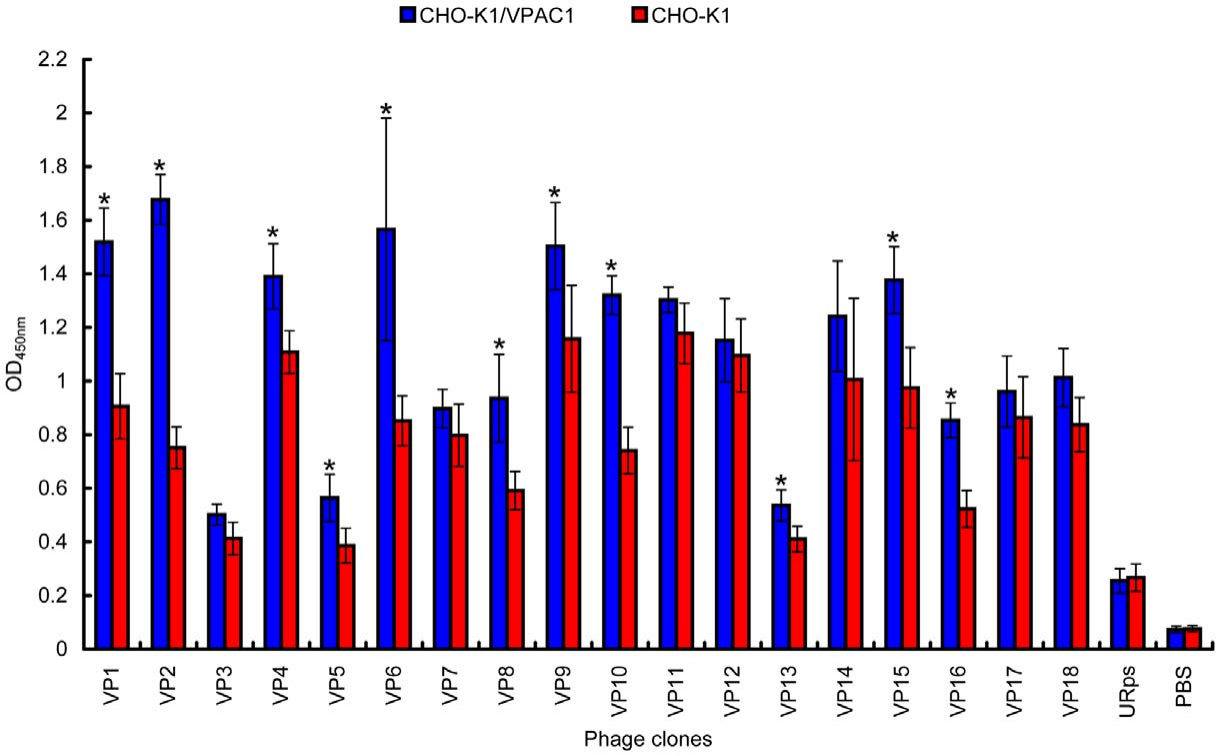
Identification of the binding selectivity of the 18 clones by cellular ELISA. Phage clones binding to CHO-K1/VPAC1 cells (*blue bars*) and wild-type CHO-K1 cells (*red bars*) were detected by the HRP-conjugated anti-M13 phage antibody. PBS and URps (unrelated phage, an amplified phage randomly selected from the original phage peptide library) were used as negative controls. Triplicate determinations were done at each data point, and average OD_450 nm_ of two types of cells are shown. Single asterisk denotes *p*<0.01(OD_450 nm_ of each clone binding to CHO-K1/VPAC1 cells versus CHO-K1 cells).

A cellular ELISA was performed to determine the affinity of the 18 phage clones for CHO-K1/VPAC1 cells and exclude false positives and clones that bound with equal affinity to CHO-K1/VPAC1 and CHO-K1 cells. To determine the selectivity, the affinity of each clone for CHO-K1/VPAC1 cells was compared to its affinity for wild-type CHO-K1 cells. The results showed that phages VP1, VP2, VP5, VP6, VP8, VP10 and VP16 appeared to bind with higher affinity to CHO-K1/VPAC1 cells than CHO-K1 cells. In contrast, the URps (unrelated phages) bound similarly and with low affinity to the two types of cells (Figure 3). Among the 7 positive phage clones, VP2 bound most effectively. Therefore, the phage clone VP2 and its displaying peptide were further investigated.

### Competitive inhibition assay

**Figure 4:**
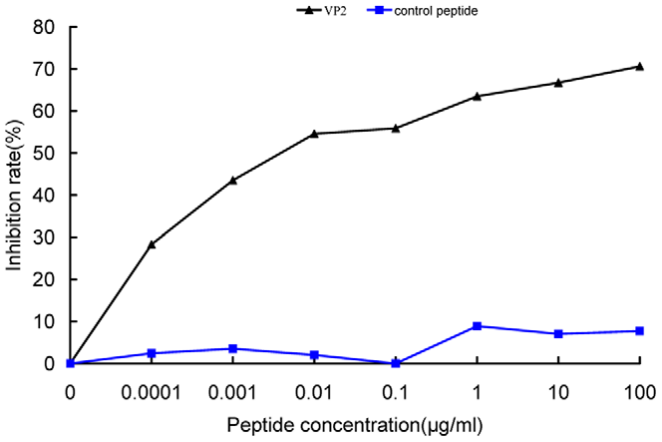
Competitive inhibition of binding of the positive phage clone VP2 to CHO-K1/VPAC1 cells by the synthetic peptide VP2. The average inhibition rates at different concentrations of the VP2 peptide were shown. When the concentration of VP2 peptide was increased above 0.001 µg/ml, a significant inhibition occurred. An unrelated peptide displayed by the unrelated phage was used as a negative control.

The peptide-competitive inhibition assay was performed to determine whether the synthetic peptide VP2 (GFRFGALHEYNS) and the corresponding positive phage clone could compete for the same binding site. Our results demonstrated that when synthetic VP2 peptide was pre-incubated with CHO-K1/VPAC1 cells, the binding of the positive phage clone VP2 was inhibited in a dose-dependent manner, demonstrating that the positive phage clone bound to CHO-K1/VPAC1 cells by displaying the VP2 peptide. When the concentration of exogenous VP2 peptide was increased, the number of positive VP2 phages binding to CHO-K1/VPAC1 cells decreased, and the rate of inhibition increased gradually. When the peptide concentration was increased above 0.001 µg/ml, significant inhibition occurred, and the IC_50_ was approximately 18.5 µg/L (13.2 nM) (Figure 4). A control peptide (an unrelated peptide displayed by an unrelated phage) had no effect on the binding of VP2 phage to CHO-K1/VPAC1 cells.

### Binding specificity of the VP2 peptide to the VPAC1 receptor

**Figure 5:**
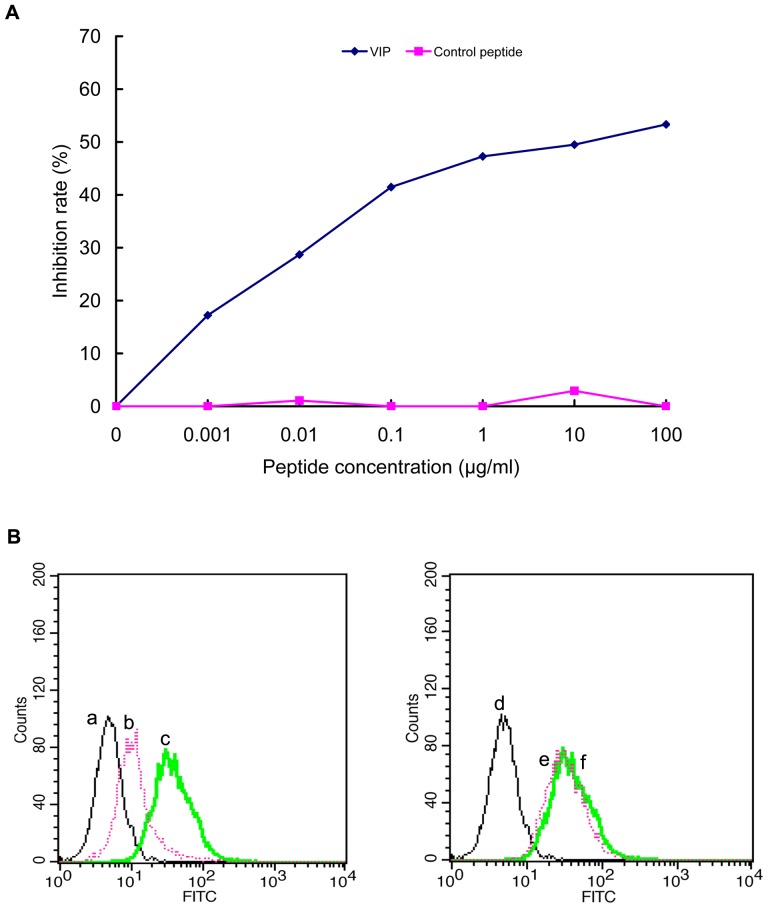
Binding specificity of the VP2 peptide to the VPAC1 receptor. (A) Competitive inhibition ELISA by VIP. The average inhibition rates at different concentrations of VIP were shown. When the concentration of VIP was increased above 0.001 µg/ml, a significant inhibition occurred. An unrelated peptide displayed by the unrelated phage was used as a negative control. (B) Flow cytometry analysis of the inhibition effect of VIP on binding of VP2 peptide to CHO-K1/VPAC1 cells. Here, a,d: blank control, b: VIP+FITC-VP2, e: Unrelated peptide+FITC-VP2, c,f: FITC-VP2.

To investigate the effect of the positive phage clone and its corresponding peptide VP2 on the binding of the VPAC1 receptor to its native ligand VIP, two competitive inhibition experiments were performed. The results of a competitive inhibition ELISA showed that with an increase in the concentration of VIP, the number of VP2 phages binding to CHO-K1/VPAC1 cells decreased, the rate of inhibition increased gradually, and the IC50 was approximately 9.1 µg/ml (2.7 µM) (Figure 5A). Because the positive phage clone bound to CHO-K1/VPAC1 cells through the peptide VP2, VIP and VP2 could compete for the same binding site on the VPAC1 receptor. A control peptide had no effect on the binding of the positive phage clone to CHO-K1/VPAC1 cells.

Flow cytometry analysis was performed to evaluate the binding specificity of the FITC-VP2 peptide. We found that when incubated together, the FITC-VP2 peptide bound specifically to CHO-K1/VPAC1 cells (Figure 5B). When VIP was incubated with CHO-K1/VPAC1 cells, the binding activity of FITC-VP2 was significantly inhibited, indicating that VIP had a negative effect on FITC-VP2 binding to CHO-K1/VPAC1 cells (Figure 5B). These results further confirmed that VIP and VP2 peptides could compete for the same binding site, and VP2 specifically bound to the VPAC1 receptor. When an unrelated peptide was incubated with CHO-K1/VPAC1 cells, it had no effect on the binding of FITC-VP2 to these cells (Figure 5B).

### Binding of VP2 to CHO-K1/VPAC1 and colorectal cancer cell lines

**Figure 6:**
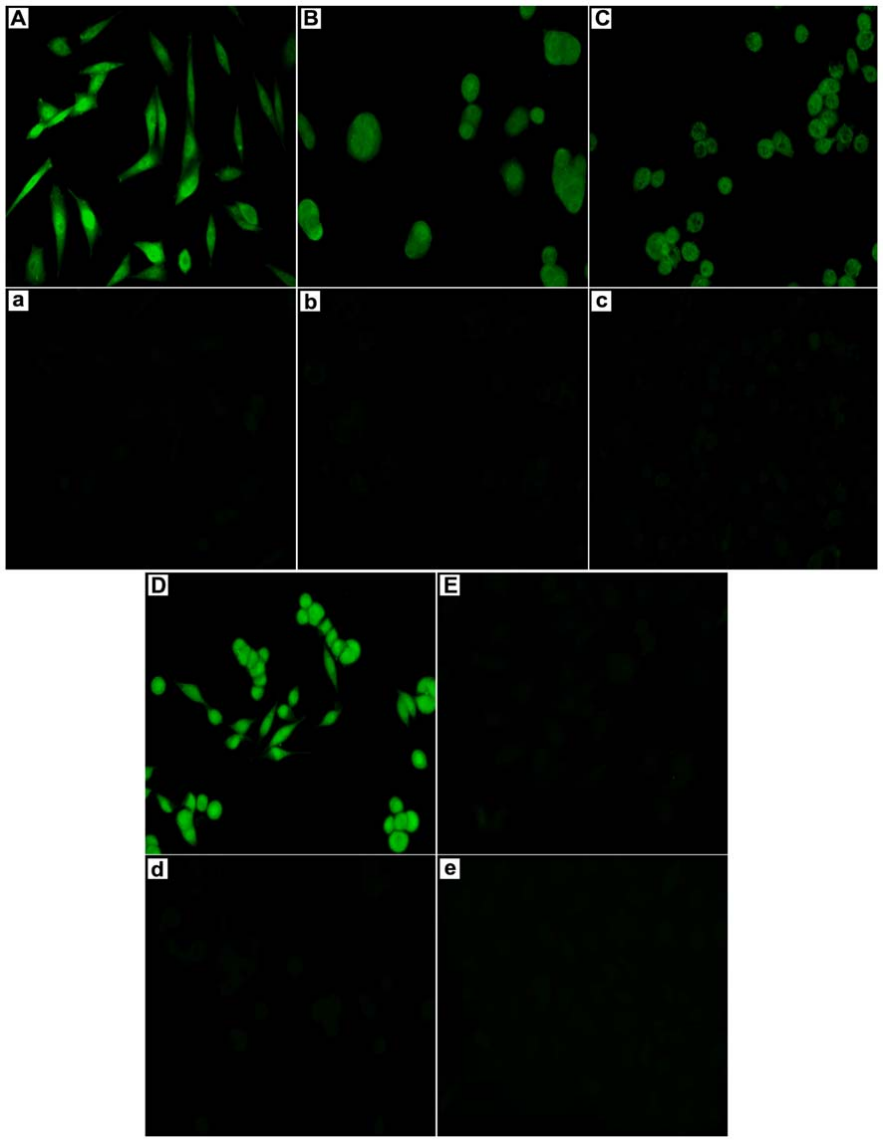
Binding of VP2 peptide to CHO-K1/VPAC1 cells and colorectal cancer cell lines (×200). The FITC-conjugated VP2 (FITC-VP2) was incubated with CHO-K1/VPAC1 (A), HT29 (B), SW480(C), SW620 (D) and control CHO-K1 cells (E). At the same time, the control FITC-conjugated unrelated peptide (FITC-URp) was incubated with CHO-K1/VPAC1 (a), HT29 (b), SW480 (c), SW620 (d) and CHO-K1 cells(e). The cells were observed under a fluorescence microscope.

**Figure 7:**
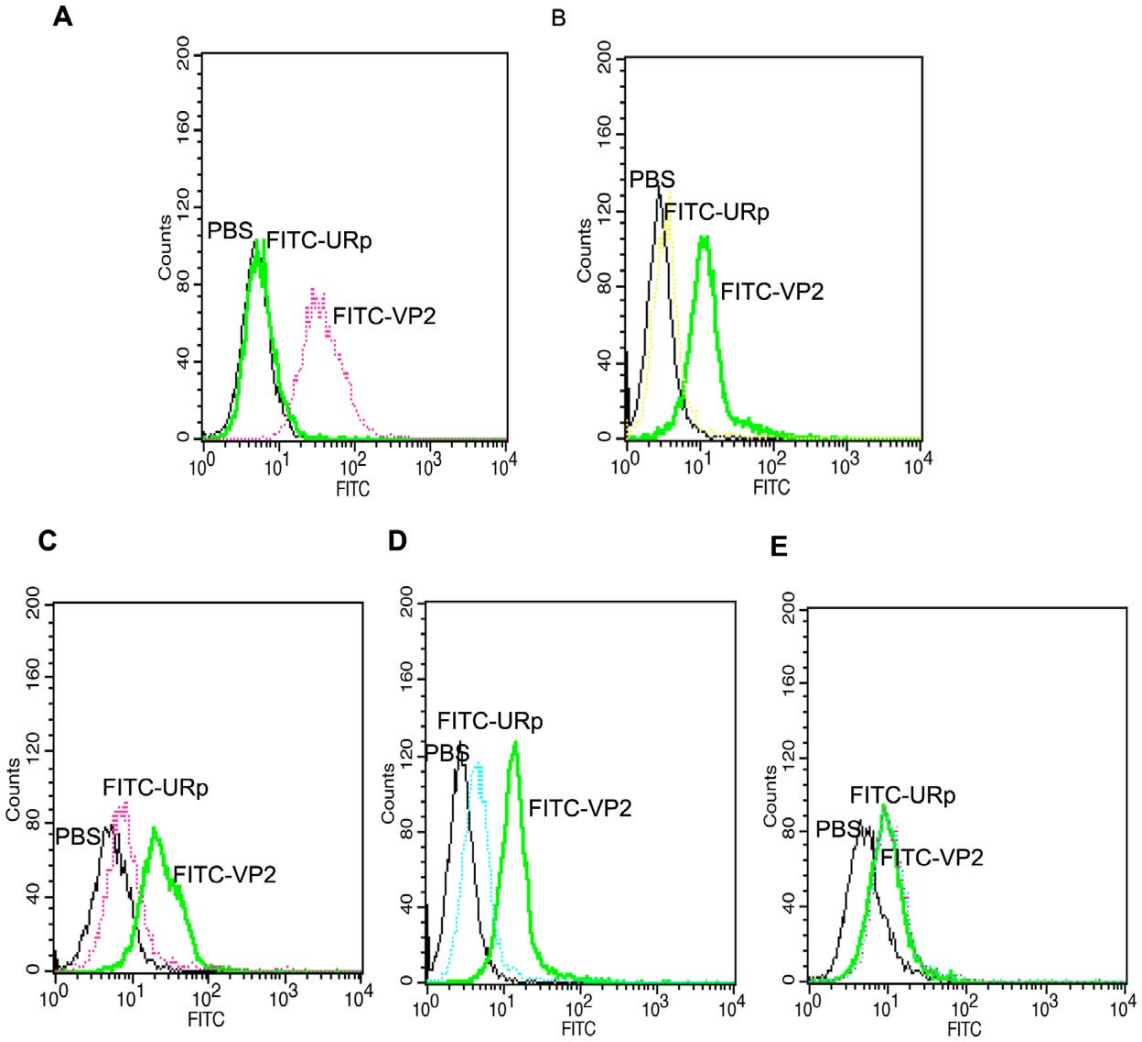
FACS analysis of VP2 peptide binding to CHO-K1/VPAC1 cells and colorectal cancer cell lines. The FITC-VP2 and the control FITC-URp were incubated with CHO-K1/VPAC1(A), HT29(B), SW480(C), SW620 (D) and control CHO-K1 cells(E), respectively. PBS was used as a blank control. Triplicate determinations were done at each data point.

The results of the experiments described above demonstrate that the VP2 peptide can specifically bind to the VPAC1 receptor. To directly observe the binding of VP2 to CHO-K1/VPAC1 cells and further investigate whether VP2 could bind to CRC cells that express VPAC1 receptors at high levels, a fluorescence microscopy assay using FITC-conjugated VP2 (FITC-VP2) was performed. After CHO-K1/VPAC1, HT29, SW480, SW620 and CHO-K1 cells were incubated with FITC-VP2, specific fluorescence was observed on the membrane and in the perinuclear cytoplasm of CHO-K1/VPAC1, HT29, SW480 and SW620 cells using a fluorescence microscope. In contrast, there was no significant green fluorescence in the control CHO-K1 cells, and negative results were obtained in all cell types when a FITC-conjugated control peptide was used in place of FITC-VP2 (Figure 6). Flow cytometry analysis indicated that the fluorescence intensities of CHO-K1/VPAC1, HT29, SW480, and SW620 cells incubated with FITC-VP2 were 87.1±4.1 (Figure 7A), 68.9±3.1 (Figure 7B), 63.4±3.5 (Figure 7C), and 77.8±4.2 (Figure 7D), respectively, and the corresponding fluorescence intensities observed when the cells were incubated with a FITC-labeled unrelated peptide (FITC-URp) were 3.4±0.4 (Figure 7A), 3.9±0.4 (Figure 7B), 4.3±0.5 (Figure 7C), and 4.8±0.7 (Figure 7D), respectively (*p*<0.01). The fluorescence intensities of control CHO-K1 cells incubated with FITC-VP2 and FITC-URp were 3.6±0.7 and 4.4±0.7 (Figure 7E), respectively (*p*>0.05). These results suggest that VP2 peptide binds specifically to CHO-K1/VPAC1 cells and several types of colorectal cancer cell lines.

## Discussion

CRC, a predominant gastrointestinal malignancy, has been the second most common cause of cancer-related deaths over the past several years [28]. In the progressive stage, CRC is strongly invasive, has a high post-operative recurrence rate and is difficult to cure [29]. When treated in the early stages, CRC patients can achieve relatively positive outcomes; thus, early detection is crucial for reducing CRC mortality. Cancer cells often display high numbers of certain cell surface molecules, such as tumor-associated antigens or specific receptors, that infrequently occur in normal tissues and represent potential targets for tumor diagnosis and treatment. In our previous study, we first reported that testes-specific protease 50 (TSP50) was abnormally and highly expressed in CRC and represented a potential effective predictor of poor prognosis in CRC patients, making it an attractive novel target for molecular imaging and therapy [30]. Several previous reports demonstrated that the VPAC1 receptor is highly expressed in CRC and plays a major role in the progression of CRC [11], [14], making it one of the most promising novel candidate markers for early CRC detection. Therefore, the screening and identification of peptides that specifically bind to the VPAC1 receptor will aid the development of novel probes for CRC detection and therapy. For this purpose, we utilized a phage display peptide library, and to the best of our knowledge, this is the first time that the VPAC1 receptor has been used as a target to screen a 12-mer phage display peptide library in an attempt to obtain peptides that specifically bind to the VPAC1 receptor.

The most common screening strategy involves purifying a specific target, absorbing it to an affinity resin or ELISA plate, and screening for binding by adding a phage peptide library [31], [32]. This method requires further confirmation of binding using the native form of the target in cells or tissues because peptides selected using purified recombinant protein might not be capable of accessing their targets in living cells. Our panning strategy employed intact and viable cells stably expressing VPAC1 receptors as target cells, which ensures a more specific target [33], [34] and the isolation of a peptide that can specifically bind to cells expressing VPAC1 receptors. To decrease non-specific binding in each round, the original phage library was panned against absorber CHO-K1 cells before screening with CHO-K1/VPAC1 cells. During the panning process, the temperature was set at 37°C when the phage library was incubated with the absorber CHO-K1 cells to ensure a minimal number of non-specific phages. When the phages were incubated with the target CHO-K1/VPAC1 cells, the temperature was changed to 4°C to rigorously select internalized phages, which represents a deviation from many other panning procedures [24], [26]. After four rounds of biopanning, the phage recovery rate gradually increased, and positive phage clones were effectively enriched, while the phage input titer was maintained (Figure 2). Additionally, after acid elution in the fourth round, a specific elution with VIP was performed to recover as many specific positive phage clones as possible.

In this study, we randomly selected 60 phage clones from three fractions of recovered phages for further characterization after the fourth round of biopanning. We performed DNA sequencing of the selected phage clones prior to cellular ELISA, which helped to eliminate some unnecessary tests because many phage clones displayed the same sequence. After the 60 phage clones were sequenced, 18 different phage clones or peptide sequences were obtained (Table 1). Subsequently, the 18 phage clones were further tested using cellular ELISA to confirm their specific binding to CHO-K1/VPAC1 cells in vitro. Among these 18 phage clones, VP2 appeared to have the highest OD_450 nm_ and selectivity value, and it bound more effectively than any of the other clones (Figure 3). Therefore, the phage clone VP2 and its displaying peptide were selected for further investigation. A multiple sequence alignment showed that the sequence did not exhibit homology to the sequences of any characterized proteins in various protein databases. This finding demonstrates that VP2 is a novel peptide and may mimic a complex epitope, which may explain why VP2 is not present in any database. Therefore, VP2 warrants further investigation regarding its biological characteristics. The results of a competitive inhibition assay demonstrated that the phage VP2 binds to CHO-K1/VPAC1 cells via the VP2 peptide (Figure 4). Although the VP2 peptide specifically binds to CHO-K1/VPAC1 cells, future studies should be performed to determine whether the peptide can specifically bind to the VPAC1 receptor. The results of two receptor-binding assays showed that the binding activity of VP2 was significantly inhibited when VIP was incubated with CHO-K1/VPAC1 cells, which also demonstrated that VIP negatively affects VP2 binding to CHO-K1/VPAC1 cells (Figure 5). These results confirmed that VIP and VP2 compete for the same binding site, further indicating that VP2 may specifically bind to the VPAC1 receptor. The VP2 peptide inhibited the binding of phage VP2 to CHO-K1/VPAC1 cells with an IC_50_ of approximately 13.2 nM (Figure 4), which was significantly lower than the IC_50_ of VIP (Figure 5A). This finding may indicate that VP2 binds to the VPAC1 receptor with a higher affinity than VIP. A fluorescence microscopy assay was performed to directly observe the binding of VP2 to CHO-K1/VPAC1 cells and CRC cells. The results of this assay indicated that VP2 specifically bound to these cells and not the control CHO-K1 cells (Figure 6). Most importantly, the peptide not only bound to the membrane but was also internalized into CRC cells; thus, this peptide could be used as a targeting vector for radionuclide or chemotherapeutic agents and is potentially valuable for targeted imaging and the treatment of CRC. The results of flow cytometry, which were consistent with the results of the fluorescence microscopy assay, further confirmed that VP2 can specifically bind to CHO-K1/VPAC1 cells and several types of CRC cell lines based on high levels of fluorescence, which likely varied because of the different expression patterns of VPAC1 receptors on these cells (Figure 7). These results indicate that VP2 binds specifically to the VPAC1 receptor and CRC cell lines and may be a useful targeting probe for the diagnosis and treatment of CRC.

VIPRs are highly expressed in CRC and play important roles in cancer-associated progression and angiogenesis, and previous studies have revealed that VIPR-targeted imaging agents coupled with radionuclides can be used for the detection of CRC [35]–[39]. Among these molecular probes, VIP and its analogues were mainly used for targeted imaging. Here, we have identified a new peptide, VP2, that can bind specifically to the VPAC1 receptor and CRC cells, indicating its potential application in the molecular imaging of CRC. Moreover, we hypothesized that this novel peptide would exhibit enhanced tumor-targeted characteristics and generate a better image when used in molecular imaging because it selectively binds to one subtype of VIPR with a higher affinity than VIP. To test our hypothesis, we radiolabeled the VP2 peptide with ^99m^Tc and performed in vivo pharmacokinetics and tumor imaging experiments in tumor-bearing nude mice. The results of these experiments indicate that VP2 may be a useful diagnostic molecular reagent for detecting CRC (data unpublished). Therefore, the development of a specific imaging probe targeting the VPAC1 receptor may provide a sensitive tool for the early detection of CRC, and as a result of early detection, CRC may be treated more successfully.

Because tumors overexpress certain receptors (e.g., the VPAC1 receptor), these receptors can be used for the receptor-targeted delivery of chemotherapeutic reagents [40]. Several reports have revealed that VIP conjugated with different types of chemotherapeutics is internalized by cancer cells expressing high levels of VPAC1 receptors and subsequently metabolized by proteolytic enzymes, which results in a release of the cytotoxic chemotherapeutics within the cells and effectively leads to cancer cell death [41]–[43]. Based on these findings, we suppose whether the VP2 peptide can serve as a VPAC1 receptor-targeted vector for the delivery of cytotoxic antitumor drugs due to its specificity for the VPAC1 receptor and its internalization by CRC cell lines. Furthermore, previous studies have indicated that the VIP-VPAC1 receptor system plays a vital role in the growth, progression and angiogenesis of multiple tumors [13], [14], [16], [44]. Thus, the interruption of VIP signaling with specific antagonists represents a new therapeutic approach for the treatment of cancers. And several VIP receptor antagonists were already identified that could significantly inhibit the clonal growth of cancer cells and reduce tumor volumes [14], [45], [46]. Because VP2 is a novel peptide, it will be necessary to determine whether VP2 can act as an antagonist to the VPAC1 receptor and understand the biological function of VP2. In view of this, bioinformatics analysis also provides a new method of examining the effects of sequence and structural alterations of a given peptide to improve its stability and in vivo dynamics such that the most effective form of the peptide can be utilized in clinical and therapeutic fields. Therefore, additional research examining whether the VP2 peptide can act as a vector facilitating the transfer of chemotherapeutics or act as an inhibitor of the VPAC1 receptor expressed in CRC and other cancer cell lines will be performed with the aim of developing VP2 as a novel targeting probe for the treatment of CRC and other tumors.

Here, we report the selection of a peptide specific for the VPAC1 receptor using a phage display peptide library. Our results indicate that the VP2 peptide could specifically bind to VPAC1 receptor with high affinity. Experiments evaluating the characteristics of the selected peptide labeled with radionuclide as an imaging probe are currently underway, and the results of which indicate that VP2 may serve as a useful diagnostic molecular imaging probe for detecting CRC (data unpublished). Further studies should aim to identify whether VP2 can deliver anti-tumor drugs to tumors and determine whether the VP2 peptide can act as an antagonist to inhibit cancer migration and growth in animal tumor models. This study provides a basis for the further development of peptide ligand-based human VPAC1 receptor-targeted tumor diagnosis and treatment.

## Materials and Methods

### Reagents

The Ph.D.-12™ phage display peptide library kit containing E.coli host strain ER2738 was purchased from New England BioLabs (Ipswich, MA, USA). G418 was obtained from Invitrogen Biotechnology Company (Shanghai, China). The monoclonal anti-VPAC1 antibody was from Santa Cruz Biotechnology (CA, USA). PrimeScript RT reagent Kit was purchased from Takara (Dalian, China). Fluorescein isothiocyanate (FITC)-labeled goat anti-rabbit IgG was obtained from ZSGB-BIO (Beijing, China). X-gal was purchased from Amresco (Solon, OH, USA). IPTG was from Merck (Darmstadt, Germany). Protease inhibitor was purchased from Roche (Shanghai, China). Horseradish peroxidase (HRP)-conjugated anti-M13 monoclonal antibody was obtained from GE Healthcare (Piscataway, NJ, USA). Bacteria culture media, Bactotryptone and Bacto-yeast extract were purchased from OXOID (Basingstoke, Hampshire, UK). Dulbecco's modified Eagle media (DMEM), fetal bovine serum (FBS) and trypsin were purchased from Hyclone (MA, USA).

### Cell lines and cell culture

Chinese hamster ovary cells (CHO-K1 cells) and CRC cell lines HT29, SW480 and SW620 were obtained from the American Type Culture Collection (ATCC). CHO-K1, CHO-K1/VPAC1, SW480 and SW620 cells were maintained in Dulbecco's modified Eagle media (high glucose) supplemented with 10% fetal bovine serum (FBS), 100 U/ml penicillin, and 100 µg/ml streptomycin. HT29 cells were maintained in DMEM/F12 supplemented with 10% FBS, penicillin, and streptomycin. The cells were cultured at 37°C in a humidified atmosphere containing 5% CO_2_.

### Construction and identification of the CHO-K1/VPAC1 cell line

The coding region of the VPAC1 gene was subcloned into the vector pcDNA3.1 (+), which contains the selectable neomycin gene, with *HindIII* and *EcoRI* (Sangon Biotech, Shanghai, China). CHO-K1 cells were cultured in the medium mentioned above. Various concentrations of G418 were added to the CHO-K1 cells to determine the optimal selection concentration. The recombinant plasmid was then transfected into CHO-K1 cells following the manufacturers' instructions (Invitrogen Biotechnology Company, Shanghai, China). After 48 hours of recovery, G418 was added to the medium for selection (final concentration of 700 µg/ml), and the cultures were incubated for 2 weeks. Resistant clones were obtained via limiting dilution and maintained in the above-mentioned culture medium containing 200 µg/ml G418 at 37°C in the presence of 5% CO_2_. The expression of the VPAC1 receptor was measured by reverse transcription PCR, immunofluorescence and western blotting using previously reported methods [30]. Briefly, reverse transcription polymerase chain reaction was performed using specific primers (forward 5′-CCCATTGCCTGTGGTTTG-3′ and reverse 5′-CCTGGAAAGACCCCACGAC-3′) to evaluate VPAC1 gene expression in the transfected cells. A monoclonal anti-VPAC1 antibody was used in immunofluorescence and western blot experiments to evaluate the expression of VPAC1 protein. Finally, CHO-K1 cells stably expressing VPAC1 receptors were maintained in culture medium containing 200 µg/ml G418.

### In vitro subtractive panning

CHO-K1/VPAC1 and CHO-K1 cells were cultured in DMEM (high glucose) containing 10% FBS at 37°C in a humidified atmosphere containing 5% CO_2_. CHO-K1/VPAC1 and CHO-K1 cells were used as target cells and absorber cells, respectively, for a whole cell subtractive screening using a 12-mer phage display peptide library. In vitro screening procedures were performed as described in the instruction manual of the kit, with some modifications. Briefly, when the CHO-K1 cells reached 85% confluency, the culture medium was removed. The cells were washed twice with PBS and cultured with serum-free medium containing 1% bovine serum albumin (BSA) for 2 h to clear the surface receptors. Subsequently, the CHO-K1 cells (1×10^7^) were harvested using 0.25% trypsin and blocked for 30 minutes at 37°C with 5% PBS-BSA. Approximately 2×10^11^ pfu phage and 500 µl of protease inhibitor were added to the cells and incubated at 37°C for 1.5 h with gentle rotation. During this time, the CHO-K1/VPAC1 cells were pre-cleared, harvested and blocked in the same manner. After the incubation, the cells were pelleted at this and subsequent pannings by centrifugation at 1500 rpm for 2 min. The supernatant was collected, and the CHO-K1 cells (and the phages bound to them) were removed by centrifugation. The supernatant containing phages was incubated with the blocked CHO-K1/VPAC1 cells (5×10^6^) at 4°C for 1 h under slight vibration, and subsequently, the cells were pelleted again. The CHO-K1/VPAC1 cells were washed twice with 0.1% TBST (50 mM Tris-HCl, 150 mM NaCl, 0.1% Tween-20, pH 7.5) and once with 1% PBS-BSA to remove the unbound phages. Next, the cell membrane-bound phages (Mps) were eluted with 2 ml of elution buffer (0.2 M glycine-HCl, pH 2.2, 1 mg/ml BSA) for 8 min on ice and neutralized with 300 µl of 1 M Tris-HCl (pH 9.1). The elution buffer was centrifuged again, and the supernatant was collected. The cells in the precipitate were washed once with PBS-BSA and lysed with lysis buffer (2 ml of 0.1% triton, 500 µl of protease inhibitor) for 30 min at room temperature. Finally, the internalized phages (INps) contained in the cell lysate were recovered. A total of 10 µl of Mp and INp was used for titer evaluation by the blue plaque-forming assay on LB/IPTG/X-gal plates, and the remaining phages were amplified, purified and titered again. Subsequently, 1×10^11^ pfu Mp and 1×10^11^ pfu INp were mixed together and subjected to the next round of panning. Four rounds of selection were performed as described above with the following minor modifications. The concentration of TBST was increased to 0.5%, the washing frequency was altered to 7 times, the incubation time with CHO-K1 cells was increased to 2 h, and the incubation time with CHO-K1/VPAC1 cells was decreased to 30 min in a stepwise manner. The phage titers were determined after each round, and the recovery rates were calculated. After the elution step in the fourth round, CHO-K1/VPAC1 cells in the precipitate were eluted with 3 ml of 30 µM vasoactive intestinal peptide (VIP), a native ligand of the VPAC1 receptor, for 30 min at room temperature and subsequently lysed as above. We recovered three types of phages: an acid elution fraction (Mp), a specific elution fraction (Sp), and a lysate fraction (INp).

### DNA sequencing of the selected phages

After the fourth round of panning, 60 phage clones (20 Mp, 20 Sp and 20 INp) were randomly selected, amplified and purified. ssDNA was extracted according to the instruction manual of the kit. DNA sequencing was performed by BGI (Beijing, China) using the -96gIII primer (5′-CCCTCATAGTTAGCGTAACG-3′). Homologous analysis was then performed, and phages with the same sequence were classified.

### Cell enzyme-linked immunosorbent assay

CHO-K1/VPAC1 cells and control CHO-K1 cells were cultured in DMEM (high glucose) at 37°C and plated in 96-well plates (1×10^6^ cells/well) until they adhered as a monolayer. Subsequently, the cells were washed and fixed with 0.25% glutaral for 15 min at room temperature, and the plates were washed three times with PBS. A solution of hydrogen peroxide (3%; 100 µl) was added to each well, and the plates were placed in a 37°C incubator for 30 min to inhibit the activity of endogenous peroxidase. Following the incubation, the plates were washed three times with PBS, and the wells were blocked with 200 µl of 2% BSA for 30 min at 37°C. The phages were added to CHO-K1/VPAC1 and CHO-K1 cells (1×10^10^ pfu/well) and incubated at 37°C for 2 h. Subsequently, unbound phages were removed by washing the plates three times with 0.05% TBST for 5 min. In total, 100 µl of horseradish peroxidase (HRP)-conjugated anti-M13 monoclonal antibody (1∶5000) was added to each well, and the plates were incubated at 37°C for 1 h. After washing three times with 0.05% TBST, tetramethylbenzidine (TMB) was added to the wells and incubated at room temperature for 30 min. The reaction was terminated by the addition of 50 µl of 2 M H_2_SO_4_. Subsequently, the plates were read on an automated ELISA plate reader at a wavelength of 450 nm. PBS and unrelated phages (URps, amplified phages from the original phage peptide library) with equal titers were added to the wells in place of the selected phage clones to serve as negative controls. Two wells were used for each clone, and all experiments were performed in triplicate. The average A values and selectivity were calculated. Selectivity was determined using the following formula: selectivity = (OD_A1_−OD_C1_)/(OD_A2_−OD_C2_), where OD_A1_ and OD_C1_ represent the A values obtained when selected phages and unrelated phages, respectively, were incubated with CHO-K1/VPAC1 cells, and OD_A2_ and OD_C2_ represent the A values obtained when selected phages and unrelated phages, respectively, were incubated with CHO-K1 cells.

### Peptide synthesis

The candidate peptides were synthesized by ChinaPeptides (Shanghai, China) using standard solid-phase Fmoc chemistry. FITC was conjugated to the N-terminus of each candidate peptide. The products were purified to a minimum purity of 95% by high-performance liquid chromatography (HPLC) and isolated by lyophilization. The sequence and structure of each peptide were characterized by mass spectrometry, and the purity of the peptides (95%) was determined by analytical HPLC. To serve as a control, a peptide displayed on an unrelated phage was synthesized in the same manner.

### Competitive inhibition assay

Competitive binding inhibition between the positive phage clone and its encoded peptide was evaluated in this study using the competitive inhibition ELISA method. The procedure was similar to that of the cellular ELISA mentioned above in “Cell enzyme-linked immunosorbent assay”. Briefly, CHO-K1/VPAC1 cells were incubated with 100 µl of synthetic peptide encoded by the positive phage clone at various concentrations (0, 0.0001, 0.001, 0.01, 0.1, 1, 10 and 100 µg/ml) in a 96-well plate after it was blocked with 2% BSA. Subsequently, 1×10^10^ pfu homologous positive phage clones was added to the cells and incubated at 37°C for 2 h. The unbound phages were washed away, and an HRP-conjugated anti-M13 monoclonal antibody was used to detect the bound phages as described above. The rate of inhibition was calculated using the following formula: rate of inhibition = (A value of inhibition control – A value of inhibition)/A value of inhibition control×100%. An unrelated peptide corresponding to an unrelated phage was used as a negative control.

### Binding specificity of the VP2 peptide for the VPAC1 receptor

Two assays were performed to evaluate the receptor binding characteristics of the synthetic peptide VP2: competitive inhibition ELISA and flow cytometry. The competitive inhibition ELISA was performed similar to the method mentioned above, except that the peptides incubated with CHO-K1/VPAC1 cells were replaced by VIP at various concentrations (0, 0.001, 0.01, 0.1, 1, 10 and 100 µg/ml). The A values and rate of inhibition were obtained as above to demonstrate whether VIP and its positive phage clone could competitively bind to the CHO-K1/VPAC1 cells. An unrelated peptide was used as a negative control.

CHO-K1/VPAC1 cells were cultured as usual, digested, centrifuged at 1000 rpm for 5 min and washed twice in an isotonic PBS buffer (supplemented with 0.5% BSA). The cells were blocked with 2% BSA for 30 min at room temperature, centrifuged again and resuspended in PBS to a final concentration of 2×10^6^ cells/ml. Subsequently, 1 ml of cell suspension was transferred to a tube, VIP was added to a final concentration of 50 µM, and FITC-conjugated synthetic VP2 peptides were added at a final concentration of 10 µM. The tubes were then incubated for 1 h at room temperature. Unreacted FITC-conjugated synthetic VP2 peptides were removed by washing the cells twice with PBS. The cells were then resuspended in 300 µl of PBS for flow cytometry analysis. An unrelated peptide was used as a negative control.

### Fluorescence microscopy and flow cytometry

Fluorescence microscopy was used to directly observe the binding of synthetic VP2 peptide to CHO-K1/VPAC1 cells and several CRC cell lines. CHO-K1/VPAC1, HT29, SW480, SW620 and CHO-K1 cells were digested with 0.25% trypsin and plated on coverslips overnight, respectively. The cells were washed three times with PBS, cultured with serum-free medium for 1 h, and fixed with 4% paraformaldehyde at room temperature for 20 min. The cells were then washed three times with PBS and blocked with 2% BSA for 30 min. The FITC-conjugated synthetic VP2 peptides were incubated with the cells for 1 h at 37°C. After three washes, DAPI was used to stain the nucleus, and the slides were observed using fluorescence microscopy. Unrelated peptides labeled with FITC were used as negative controls. The binding ability of synthetic VP2 peptide was further verified by flow cytometry using a procedure similar to the one described above, except that VIP was not added to these cells. Finally, the cells were resuspended in 300 µl of PBS for flow cytometry analysis.

### Statistical analysis

The data were expressed as the mean ± standard deviation (SD). Statistical analysis of the data was performed using SPSS 17.0 (SPSS, Chicago, IL, USA). Statistical significance was determined using Student's *t*-test. The Wilcoxon signed rank test was employed for non-parametric analysis. A value of *p*<0.01 was considered statistically significant.

## Supporting Information

Dataset S1 **Original full DNA sequences of the selected phage clones.** After the fourth round of panning, 60 phage clones were randomly selected, amplified and purified. ssDNA was extracted and DNA sequencing was performed using the -96gIII primer. The original full DNA sequences of the selected phage clones were shown in this dataset.
(RAR)
